# Regulation of FoxP3^+^ Regulatory T Cells and Th17 Cells by Retinoids

**DOI:** 10.1155/2008/416910

**Published:** 2008-03-19

**Authors:** Chang H. Kim

**Affiliations:** Laboratory of Immunology and Hematopoiesis, Department of Comparative Pathobiology, Purdue Cancer Center, Purdue University, West Lafayette, IN 47907, USA

## Abstract

Vitamin A has both positive and negative regulatory functions in the immune system. While vitamin A is required for normal formation of immune cells and epithelial cell barriers, vitamin A deficiency can lead to increased inflammatory responses and tissue damage. The mechanism with which vitamin A and its metabolites such as retinoids negatively regulate inflammatory responses has not been clearly defined. Recently, it has been established that retinoids promote the generation of immune-suppressive FoxP3^+^ regulatory T cells while they suppress the T cell differentiation into inflammatory Th17 cells in the periphery such as intestine. These novel functions of retinoids provide a potentially important immune regulatory mechanism. In this review, we discuss the functions of retinoids in the development of the FoxP3^+^ cells and Th17 cells, the phenotype and functions of retinoid-induced FoxP3^+^ T cells, and the impact of retinoid-induced FoxP3^+^ T cells on the immune tolerance.

## 1. INTRODUCTION

The immune system is regulated by various types of cells and the factors that are produced by these cells. The cells of the immune system sense the presence of the antigens and other signals, generated from pathogens and commensals, and respond to the stimuli in positive or negative ways. Among the immune cells, CD4^+^ T cells play central roles in regulation of immune responses by activating or suppressing immune cells and tissue cells. Naïve CD4^+^ T cells, produced in the thymus, can become Th1, Th2, or Th17 cells [1–5], which act as effector cells to stimulate the immune system to clear pathogens and tumor cells. On the other hand, regulatory T cells, exemplified by FoxP3^+^ T cells, suppress the immune system to prevent overactive responses and inflammation induced by T cells and their downstream (non-T) effector cells [6–11]. Humans and mice that do not have functional FoxP3^+^ T cells as the result of mutations in the FoxP3 gene suffer from inflammatory and autoimmune diseases and die young [12–16], which serves as the evidence for the essential roles of these T cells in maintaining immune tolerance. FoxP3^+^ T cells are made in the thymus from T cell progenitors and in the periphery from naïve CD4^+^ T cells. Naïve FoxP3^+^ T cells, made in the thymus, exclusively migrate to secondary lymphoid tissues for activation and differentiation into memory type FoxP3^+^ T cells that can migrate to B cell sites, nonlymphoid tissues, or sites of Th1 or Th2 inflammation [11, 17–19]. A number of factors in the periphery can regulate the generation or differentiation of FoxP3^+^ T cells. These are antigens, antigen-presenting cells such as dendritic cells, cytokines, and tissue-specific factors that are produced in the cells of certain tissue sites. One group of such tissue-specific factors are retinoids, which can induce FoxP3^+^ T cells with a gut-specific tissue tropism from naïve FoxP3^−^ T cells [20–26]. Reciprocally, retinoids suppress the generation of inflammatory Th17 cells. We will discuss in this review the general properties and functions of retinoids, their roles in regulation of T cell differentiation, and the impact of this process on regulation of the immune system.

## 2. SYNTHESIS, RECEPTORS, AND BIOLOGICAL FUNCTIONS OF RETINOIDS

**Figure 1:**
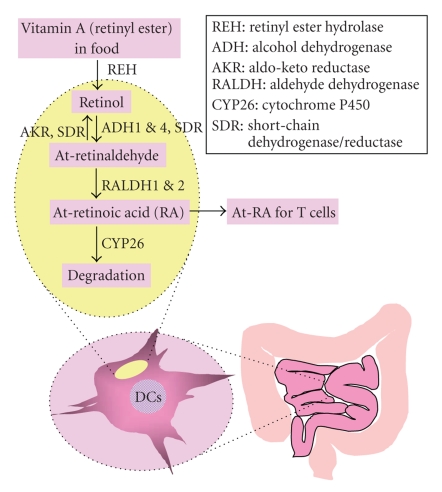
* Synthesis of retinoic acid in the dendritic cells of the intestine.* Vitamin A is consumed as retinyl ester and hydrolyzed by retinyl ester hydrolase (REH). Retinol, entered into cells, is oxidized to retinaldehyde by alcohol dehydrogenase (ADH) or short-chain dehydrogenase/reductase (SDR). The reverse reaction is mediated by aldo-keto reductase (AKR) or SDR. Retinaldehyde can be converted to retinoic acid (all-trans retinoic acid or At-RA) by retinaldehyde dehydrogenase (RALDH). Retinoic acid is degraded in cells by cytochrome P450 (CYP26). It is known that dendritic cells in the gut-associated lymphoid tissues (GALT) such as Peyer's patches and MLN express high levels of ADH1, ADH4, RALDH1, and/or RALDH2. The retinoic acid produced by dendritic cells can be exported for other cells such as T cells during the cognate interaction between dendritic cells and T cells.

Preformed vitamin A in food digesta is absorbed in the form of retinol, which can be made into retinal and retinoic acid (RA) [27] (Figure 1). Also retinol can be generated from provitamin A carotenoids such as betacarotene [28–31]. Plasma retinol binding proteins and cellular retinol binding proteins transport and stabilize retinol [32]. Some antigen-presenting cells in gut-associated lymphoid tissues have a unique feature, which is the ability to produce retinoic acid from vitamin A [33] (Figure 1). The conversion of retinol (vitamin A) to retinal is catalyzed by a subfamily of alcohol dehydrogenases (ADH), and the subsequent conversion of retinal to RA is catalyzed by retinal dehydrogenases (RALDH) [34]. While ADH5 is ubiquitously expressed by dendritic cells isolated from all of the secondary lymphoid organs, the ADH1 and ADH4 isoenzymes are preferentially expressed by only gut-associated lymphoid tissue (GALT) dendritic cells. Peyer's patch dendritic cells express RALDH1, while mesenteric lymph node dendritic cells express RALDH2 [33]. The retinoic acid, produced by GALT dendritic cells, induces T cell expression of the two gut homing receptors CCR9 and *α*4*β*7 [33]. CCR9 is the chemokine receptor for TECK/CCL25, which is expressed in the small intestine [35], and *α*4*β*7 acts as an adhesion molecule for lymphocyte rolling and firm adhesion on the surface of mucosal endothelial cells by binding to its ligand mucosal addressin cell adhesion molecule (MAdCAM-1) [36, 37].

Among the metabolites of vitamin A, 11-cis-retinal, all-trans-retinoic acid (At-RA) and 9-cis-RA mediate the major biological functions of the vitamin [29, 34]. Vitamin A has pleiotropic functions in the body. 11-cis-retinal functions as chromophores in light absorption for vision, and retinoic acid participates in bone formation, reproduction, and differentiation of many cell types during embryogenesis [28–30]. In this regard, retinoid deficiency or overdoses can cause teratogenesis [38]. Vitamin A plays important roles in the immune system. One such a function is its role in the formation of epithelial linings of the body including eyes, membranes of mucosal tissues in the respiratory, urinary and intestinal tracts, and skin, which serve as barriers to prevent the invasion by pathogens [39]. Through their effects on cell differentiation and death, retinoids inhibit or reverse the carcinogenic process in some types of cancers in oral cavity, head and neck, breast, skin, liver, and blood cells [40, 41].

**Figure 2:**
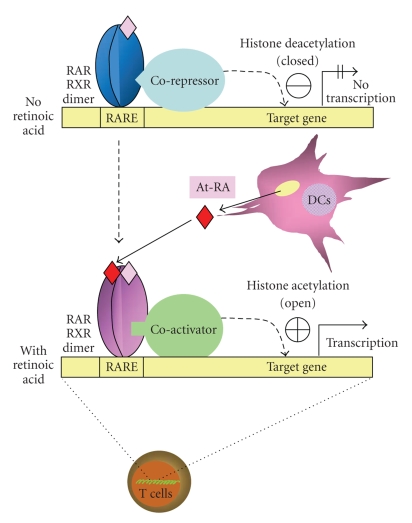
*Control of gene expression by retinoic acid*. RAR-RXR heterodimers serve as the nuclear receptors for retinoic acid. When RAR ligands (e.g., At-RA) are not available, the RAR-RXR heterodimers attract corepressors and histone acetyl deacetylases to the genes which are under the control of retinoic acid response elements (RARE: direct repeats of AGGTCA with a 5-bp spacer; also called DR-5), resulting in a closed conformation of the chromosome and blocked transcription. When RAR ligands are available in a tissue microenvironment (e.g., produced from dendritic cells in the intestine), they would enter into T cells and activate RAR-RXR heterodimers. This is followed by the release of corepressors and attraction of coactivators, which, in turn, recruit histone acetyl transferases, resulting in an open conformation of the chromosome and transcription of the genes. In this regard, the human and mouse FoxP3 promoters have several RARE's and become acetylated at histones in response to retinoic acid.

At-RA and 9-cis-RA are the ligands for retinoid nuclear receptors that act as transcription factors for gene expression. As retinoid nuclear receptors, RA receptor (RAR) isotypes (*α*, *β*, and *γ*) and the retinoid X receptor (RXR) isotypes (*α*, *β*, and *γ*) have been identified [29]. Each receptor has multiple isoforms, generated through alternative mRNA splicing and/or transcription [30, 31]. At-RA preferentially binds RARs, whereas 9-cis-RA binds equally well to both RARs and RXRs [29, 42]. The two groups of retinoid nuclear receptors form RXR/RAR heterodimers, which can function as either transcriptional repressors or activators depending on the availability and type of retinoid ligands [43]. In the absence of RAR ligands, aporeceptors recruit corepressors such as nuclear receptor corepressor (N-CoR) or silencing mediator for retinoid and thyroid hormone receptor (SMRT), which then recruit histone deacetylases (HDACs). Binding of RAR agonists to the receptors induces active conformation of the receptors (Figure 2). This decreases the affinity for corepressors and creates a binding surface for histone acetyltransferase (HAT) coactivators, such as CREB-binding protein (CBP) and p160 (e.g., TIF2). Transcriptional coactivators are involved in chromatin remodeling (decondensation due to histone acetylation) or recruitment of the basal transcription machinery. RXR agonists alone cannot dissociate the corepressors from heterodimers of RAR-RXR, and retinoid receptor antagonists would prevent the formation of the holoconformation. Please see the reviews [44, 45] for more details on retinoid receptor-mediated gene expression.

Many functions of retinoids have been identified in regulation of immune responses. As positive roles in regulation of the immune system, retinoids enhance the numbers and effector functions of neutrophils, NK cells, B cells, and Th2 cells [28, 46–52]. Retinoids induce the gut homing receptor expression in T and B cells [19, 33, 53–55] and B cell Ig switch to IgA [51, 55], and, therefore, are important regulators of mucosal immunity. Vitamin A is required for antibody responses to T-dependent and bacterial polysaccharide antigens [56, 57], normal IgA levels in the intestinal fluid [58], prevention of activation-induced T cell apoptosis [59, 60], and normal phagocytic functions and resistance to bacterial pathogens [61]. Vitamin A supplementation can decrease serum concentrations of inflammatory cytokines such as TNF-*α* and IL-1 but increase suppressive cytokines such as IL-10 [42, 62]. In this regard, supplementation with vitamin A can suppress inflammation in experimental autoimmune encephalomyelitis [47, 63–66]. Paradoxically, vitamin A deficiency is linked to both defective delayed type hypersensitivity [67–69] and excessive Th1 cell function [46–50, 70, 71]. Vitamin A is also linked to both lower and higher incidences of several types of cancers in humans [72, 73].

Retinoids are being used to treat acne, psoriasis, and selected cancer types. The mechanisms of action for the retinoids in treatment of psoriasis and acne are poorly elucidated [74, 75]. The skin cells can be the direct therapeutic target for retinoids. It is equally possible that the therapeutic effect of retinoids can be indirect though regulation of the immune system that mediates inflammation in the skin. Additionally, retinoids have therapeutic effects on acute promyelocytic leukemia (APL) cells and AIDS-related Kaposi's sarcoma cells by regulating cellular differentiation and/or proliferation [76, 77]. APL is a specific form of myelocytic leukemia characterized by the t(15;17) chromosomal translocation and formation of the abnormal PML-RAR*α* fusion protein [78, 79]. Retinoic acid, when administered at pharmacological doses, can induce leukemic cell differentiation and cell-cycle arrest by reversing the dominant-negative effects of PML-RAR*α* fusion protein on the functions of the wild-type PML and RAR*α* proteins [41, 80, 81].

## 3. REGULATION OF THE FOXP3^+^ T CELL GENERATION BY RETINOIDS

FoxP3^+^ T cells are regulatory T cells that can suppress immune responses. FoxP3^+^ T cells are made largely in two different ways: they are made in the thymus from T cell progenitors or they can be made from FoxP3^−^ naïve T cells in the periphery during their activation with antigen-presenting cells such as dendritic cells. In the periphery, many factors can affect the generation of FoxP3^+^ T cells from naïve T cells. One of the factors is the route of antigen administration. Immunization through the oral route has the tendency to induce FoxP3^+^ T cells [82, 83]. Another factor is the cytokine milieu. TGF-*β*1 is a very effective cytokine that induces FoxP3^+^ T cells from naïve T cells [84]. In this regard, the induction of FoxP3^+^ T cells by mucosal immunization is dependent on TGF-*β*1 [82, 83]. Another cytokine, IL-2, is important for peripheral induction and maintenance of FoxP3^+^ T cells [85–88]. IL-2 signaling is dispensable for the induction of FoxP3^+^ T cells in the thymus but required for maintenance of FoxP3^+^ T cells in the periphery [87, 89]. It is not just a coincidence that FoxP3^+^ T cells highly express CD25 (the alpha chain of the heterotrimeric high-affinity IL-2 receptor) to receive the IL-2 signal. Another factor is the type of antigen-presenting cells. Both monocyte-derived dendritic cells and plasmacytoid dendritic cells can induce CD4^+^ CD25^+^ T cells or FoxP3^+^ T cells [90–92]. Dendritic cells in tumors are particularly efficient in inducing FoxP3^+^ T cells [93], which provides an explanation for the high prevalence of FoxP3^+^ T cells in many types of tumors. Yet another factor is the presence of certain pathogens or toll-like receptor (TLR) ligands [94, 95]. TLR ligands can either expand FoxP3^+^ T cells or suppress their generation and functions [96, 97]. The FoxP3^+^ T cell expansion by dendritic cells can be augmented in the presence of TLR ligands such as LPS [98].

**Figure 3:**
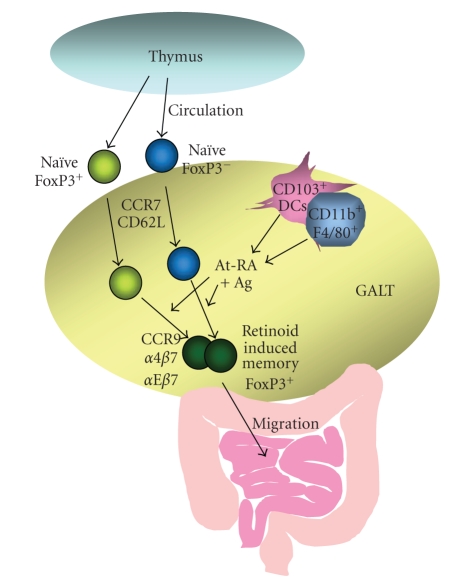
*Induction and homing of retinoid-induced FoxP3^+^ T cells*. Gut homing FoxP3^+^ T cells are made in gut-associated lymphoid tissues (GALT) but not in the thymus. FoxP3^+^ T cells made in the thymus have the naïve T cell phenotype in expression of homing receptors and migrate to secondary lymphoid tissues. In the presence of retinoic acid, these naïve FoxP3^+^ T cells become gut homing memory FoxP3^+^ T cells. Also, naïve FoxP3^−^ T cells become FoxP3^+^ T cells in the GALT to become gut homing FoxP3^+^ T cells. These FoxP3^+^ T cells express CCR9 and *α*4*β*7 among other trafficking receptors and are highly efficient in migration to the small intestine. The differentiation of these T cells into gut homing FoxP3^+^ T cells is mediated by subsets of dendritic cells and macrophages because they can present antigens and produce retinoic acid. Additionally, the epithelial cell interacting adhesion molecule *α*E*β*7 is upregulated on FoxP3^+^ T cells if the tissue microenvironments have high levels of TGF-*β*1. Intestine is such a tissue site that produces both retinoic acid and TGF-*β*1.

Recently, a number of groups including our group reported that retinoic acid regulates the peripheral induction of FoxP3^+^ CD4^+^ T cells [20–26]. Retinoic acid, together with TGF-*β*1, induces not only CD4^+^ but also CD8^+^ FoxP3^+^ T cells [20]. As mentioned before, the dendritic cells in gut-associated lymphoid tissues have the capacity to provide retinoic acid to T cells undergoing antigen priming [33]. While splenic dendritic cells could not induce FoxP3^+^ T cells from naïve T cells, the dendritic cells isolated from Peyer's patches and small intestinal lamina propria were able to induce FoxP3^+^ T cells (Figure 3) [22–24]. In this regard, Coombes et al. observed that CD103^+^ mesenteric lymph node (MLN) DCs, but not CD103^−^ MLN DCs, were efficient in conversion of naïve CD4^+^ T cells into FoxP3^+^ T cells [23]. Consistently, Sun et al. observed that the conversion rate of naïve T cells into FoxP3^+^ T cells was high in intestinal lamina propria and MLN but not PLN [22]. This conversion was dependent on TGF-*β*1 as neutralizing anti-TGF-*β*1 was able to block the induction while exogenous TGF-*β*1 further enhanced the conversion. CD103^+^ DCs express TGF-*β*2, tissue plasminogen activator, and TGF-*β* binding protein 3 [23]. Tissue plasminogen activator activates latent TGF-*β*, and TGF-*β* binding protein 3 regulates secretion and localization of TGF-*β* [99–101]. Another group independently reported that CD103^+^ DCs are CD11b^−^ and induce IL-10 but fail to induce IL-17 production in T cells [102]. Not only DCs but also some macrophages in the gut can induce FoxP3^+^ T cells: CD11b^+^ F4/80^+^ CD11c^−^ macrophages express anti-inflammatory molecules such as IL-10, TGF-*β*1, TGF-*β*3, program death-ligand 1 (PD-L1 or also called B7H-1), and PD-L2 (B7-DC), and are efficient in inducing FoxP3^+^ T cells [102]. The capacity of these antigen-presenting cells in induction of FoxP3^+^ T cells is associated with their ability to produce retinoic acid. Kang et al. showed that diethylaminobenzaldehyde (DEAB), an inhibitor of retinaldehyde dehydrogenases, completely suppressed the induction of FoxP3^+^ T cells by the mucosal DCs [24]. Mucida et al., Sun et al., and Kang et al. demonstrated that the mucosal DC-dependent induction of FoxP3^+^ T cells can be blocked by RAR antagonists such as LE540, LE135, and Ro41-5253 [20, 22, 24]. The mucosal DC-dependent induction of FoxP3^+^ T cells can be mimicked by activating naïve T cells with polyclonal T cell activators in the presence of retinoic acid and TGF-*β*1 [20–26].

FoxP3^+^ T cells of humans and mice behave somewhat differently. T cell activation readily upregulate FoxP3 in some human T cells [103, 104]. In this regard, the human FoxP3 promoter contains six NF-AT and AP-1 binding sites [104]. These molecules act as the major transcription factors that are activated downstream of TCR activation. However, the expression levels of FoxP3 in the activated T cells are relatively low and transient [105], and these activated T cells cannot suppress the proliferation of target T cells. Enforced expression of FoxP3 in human T cells resulted in induction of hyporesponsiveness and suppression of IL-2 production but failed to turn the T cells into T regulatory cells that can suppress target T cells [106], suggesting that additional signals are required to differentiate the FoxP3-expressing cells into fully functional FoxP3^+^ T regulatory cells. In contrast, simple activation of mouse FoxP3^−^ T cells in the absence of T regulatory cell-inducing factors such as TGF-*β*1 does not readily generate FoxP3-expressing T cells [20–26], implying that the expression of FoxP3 is more tightly regulated in mouse T cells. Contrary to human T cells, enforced expression of FoxP3 in mouse T cells was sufficient to turn FoxP3^−^ T cells into functional T regulatory cells which can suppress target cells [107, 108]. There is another difference between mouse and human T cells. Retinoic acid alone is sufficient to convert human naïve T cells undergoing activation into FoxP3^+^ T cells [24]. However, the TGF-*β*1 signal is additionally required to reliably induce FoxP3 in mouse T cells in vitro [20–26]. It is possible that human T cells are intrinsically different from mouse T cells in expression of certain genes such as FoxP3 as discussed above. It is also possible that human naïve T cells, but not mouse T cells, would produce TGF-*β*1 [109] at levels high enough for their conversion into FoxP3^+^ T cells in response to retinoic acid.

Retinoic acid induces histone acetylation in the human FoxP3 promoter [24]. This acetylation is thought to open the FoxP3 promoter for active transcription. It is believed that retinoic acid decreases the affinity for nuclear receptor corepressor and recruits histone acetyltransferase coactivators [110], inducing chromatin decondensation and recruitment of the basal transcription machinery to the FoxP3 promoter (Figure 2).

In a manner similar to the regular FoxP3^+^ T cells, IL-2 is required for induction of retinoid-induced FoxP3^+^ T cells [20, 24]. IL-2 at high concentrations can increase the induction of retinoid-induced human FoxP3^+^ T cells [111]. IL-2 signaling activates STAT5a and b [112], which in turn are involved in the expression of FoxP3 [113]. It has been reported that RAR*α* can interact with STAT5a and b and coregulate gene expression [114]. This implies that IL-2 signaling and STAT5 are required for the retinoic acid-induced FoxP3 expression and generation of functional T regulatory cells. This, however, turned out to be not true. Elias et al. reported that STAT5-deficient naïve T cells, although less efficient than wild-type T cells, can still be converted to FoxP3^+^ T cells in response to retinoic acid and TGF-*β*1 [26]. Therefore, the retinoic acid & TGF-*β*1-induced induction of FoxP3 expression can be enhanced by the IL-2 signaling pathway but does not absolutely require the IL-2/STAT5 signaling pathway.

The T cells stimulated by RA preferentially express RAR*α*, and RAR*α* antagonists can completely block the retinoic acid-induced expression of FoxP3 in human T cells [24, 25]. RAR*α* agonists can mimic retinoic acid in induction of FoxP3 [24, 25]. Therefore, RAR*α* is considered a central receptor that mediates the function of retinoic acid. In this regard, Schambach et al. overexpressed RAR-*α* in mouse T cells by retroviral gene transfer and observed an increase in the FoxP3^+^ T cell induction [25]. The specific role of RAR*α* is further supported by the information that methoprenic acid (a pan-RXR agonist) was hardly able to induce human FoxP3^+^ T cells [24].

In vivo, repetitive injection of retinoic acid subcutaneous induced small intestine homing FoxP3^+^ T cells in peripheral lymph nodes, which are not, normally, the sites for generation of these T cells [111]. Also, injection of RAR antagonists decreased the frequencies of FoxP3^+^ T cells in the mouse small intestine [20]. Moreover, the expression of RAR*α* itself is induced in T cells activated by retinoic acid [24], which is considered a positive feedback mechanism for generation of retinoid-induced FoxP3^+^ T cells.

## 4. REGULATION OF HOMING RECEPTOR EXPRESSION BY RETINOIDS

It is striking that the retinoid-induced FoxP3^+^ T cells in mice and humans have a highly specific tissue tropism for the small intestine. Human retinoid-induced FoxP3^+^ T cells highly express CCR9 and *α*4*β*7 and efficiently migrate toward the small intestine chemokine TECK/CCL25 [24]. On the other hand, loss of CD62L expression on the retinoid-induced FoxP3^+^ T cells would decrease their migration into peripheral lymph nodes [24] but further promote their migration into the small intestine. The mouse or human retinoid & TGF-*β*1-induced FoxP3^+^ T cells are similar to human retinoid alone-induced FoxP3^+^ T cells in high expression of CCR9 and *α*4*β*7 [24]. Interestingly, CD103 (the alpha chain of the integrin *α*E*β*7) is more highly expressed by the retinoid & TGF-*β*1-induced FoxP3^+^ T cells than TGF-*β*1 alone-induced FoxP3^+^ T cells [20, 111]. Another pathway to generate gut homing FoxP3^+^ T cells is to antigen-prime preexisting FoxP3^+^ T cells in the presence of retinoic acid (retinoid-conditioned FoxP3^+^ T cells) [19, 111]. In this case, no TGF-*β*1 is required to induce FoxP3^+^ T cells. Retinoid & TGF-*β*1-induced FoxP3^+^ T cells and retinoid alone-induced (or conditioned) FoxP3^+^ T cells would migrate to the small intestine (Figure 3) but they may localize in different locations within the small intestine due to the difference in CD103 expression.

## 5. EFFECTOR FUNCTIONS OF RETINOID-INDUCED FOXP3^+^ T CELLS

One can ask if the retinoid-induced FoxP3^+^ T cells are really regulatory T cells in terms of functionality because simple expression of FoxP3 would not necessarily indicate full maturation into T regulatory cells. In this regard, the retinoid-induced FoxP3^+^ T cells, induced by retinoic acid from naïve human T cells or by retinoic acid plus TGF-*β*1 from mouse or human T cells, appear to have all of the features of the regulatory T cells. These retinoid-induced human and mouse FoxP3^+^ T cells are hypoproliferative and can suppress the proliferation of other T cells [21, 23–25]. When injected in vivo, retinoid-induced FoxP3^+^ T cells efficiently suppressed naïve T-cell-induced colitis in recombinase-activating gene- (RAG-) deficient mice [20, 24]. The efficacy of retinoid & TGF-*β*1-treated T cells in suppression of colitis was higher than TGF-*β*1 alone-treated T cells [20, 24]. However, it is unknown if retinoid-induced FoxP3^+^ T cells are more efficient than TGF-*β*1 alone-induced FoxP3^+^ T cells in suppression of diseases in vivo because it is likely that the retinoid & TGF-*β*1-treated T cells contain more FoxP3^+^ T cells than TGF-*β*1 alone-treated T cells. Another explanation is that the retinoic acid-induced FoxP3^+^ T cells are more stable than TGF-*β*1 alone-induced FoxP3^+^ T cells in vivo even after antigenic challenge [20–22]. It has been reported that some human and mouse FoxP3^+^ T cells express granzymes and can kill target cells [115–117]. We observed a difference in expression of effector molecules between retinoid-induced human FoxP3^+^ T cells and retinoid & TGF-*β*1-induced human FoxP3^+^ T cells [24]. Retinoid-induced human FoxP3^+^ T cells express granzymes but the retinoid & TGF-*β*1-induced FoxP3^+^ T cells do not express these cytotoxic molecules because TGF-*β*1 effectively suppresses their expression [24]. While the retinoid-induced FoxP3^+^ T cells have the potential to kill target cells for immunological tolerance in the intestine, it is unknown if the FoxP3^+^ T cells, present in the intestine of mice and humans, can express granzymes and kill target cells.

## 6. REGULATION OF Th17 CELLS BY RETINOIDS

It was believed that Th1 cells were the major T cells to induce autoimmune diseases [118]. An increasing body of evidence, however, suggests that Th1 cells (and their cytokine product IFN-*γ* or transcription factor T-bet) play a protective role in certain autoimmune diseases such as experimental allergic encephalomyelitis and collagen-induced arthritis [119–121]. This implies that there are additional cell subsets that would cause autoimmune diseases, and Th1 cells would suppress the generation or function of the inflammatory cells. Many groups have reported that blocking of IL-23 or IL-17 prevented the development of autoimmune diseases [122–127]. Now, it is well established that Th17 cells constitute a newly identified subset of inflammatory T cells [3–5]. Th17 cells characteristically express IL-17A, IL-17F, IL-21, and IL-22 [128–130]. IL17A and IL-17F play important roles in inflammation and host defense by inducing IL-6, GM-CSF, G-CSF, and chemokines [131–133]. IL-21 promotes the generation of Th17 cells [134, 135]. Interestingly, IL-22 can play anti-inflammatory roles and induces antimicrobial proteins and lipopolysaccharide-binding protein [136–139]. It is well established that IL-6 and TGF-*β*1 are the major cytokines that induce mouse Th17 cells [140, 141]. In this regard, mouse Th17 cells share TGF-*β*1 with FoxP3^+^ T cells as an inductive cytokine. However, TGF-*β*1 alone or together with IL-6 does not promote the generation of human Th17 cells [142]. Also, there is an IL-6-independent pathway to generate Th17 cells [143]. IL-6-independent generation of Th17 cells is mediated, in part, by IL-21 and IL-23 [134, 135, 144]. Moreover, IL-6 induces expression of more IL-21 and IL-23 receptors on T cells to promote Th17 cell generation [144]. Important transcription factors for generation of Th17 cells include STAT3 (activated by IL-6 and IL-23), ROR-*γ*t, and ROR-*α* [145–148]. ROR-*γ*t is an orphan nuclear receptor important also for survival of CD4^+^8^+^ thymocytes and the biology of lymphoid tissue inducer cells [149–151]. ROR-*α* is another related orphan nuclear receptor, which is induced by TGF-*β*1 and IL-6 in T cells [148]. Yang et al. demonstrated that overexpression of ROR-*α* promoted Th17 differentiation, while ROR-*α* deficiency suppressed the process. Moreover, ROR-*α* and ROR-*γ*t, when overexpressed together, synergistically enhanced Th17 differentiation [148]. As negative regulators of Th17 cell generation, IL-27 (a Th1 promoting cytokine) [152, 153] and IL-2 [154] have been identified. Amadi-Obi et al., however, reported that IL-2 can expand human Th17 cells [155]. In this regard, use of IL-2 in expansion of human Th17 cells has been reported by others too [142]. Thus, whether inflammatory Th17 cells or suppressive FoxP3^+^ T cells are generated in the periphery would be determined by the cytokine milieu determined by IL-2, IL-6, IL-21, IL-27, TGF-*β*1, and other Th1/2 cytokines present in the tissue microenvironment. Additionally, there seems to be some differences in induction of Th17 cells between mice and humans.

Antigen-presenting cells would play critical roles in generation of Th17 cells because these cells can present antigens and produce certain inflammatory cytokines that can induce Th17 cells. In this regard, it was reported that MLN CD103^−^ DCs produce inflammatory cytokines such as TNF-*α*, IL-6, IL-12p40, and IL-23p19 upon stimulation with LPS [23]. While Coombes et al. did not examine if the CD103^−^ DCs could induce Th17 cells, another group examined the potential of mucosal dendritic cells in induction of Th17 cells [102]. Denning et al. reported that intestinal lamina propria CD11b^+^ DCs were able to induce Th17 cells very well, while CD11b^−^ DCs were not efficient. CD11b^+^ DCs do not express CD103, and thus they are the CD103^−^ DCs that Coombes et al. described in a separate study [23]. In contrast, CD11b^−^ DCs highly express CD103 and fail to induce IL-17 but are efficient in induction of the anti-inflammatory cytokine IL-10 and FoxP3^+^ regulatory T cells [102].

**Figure 4:**
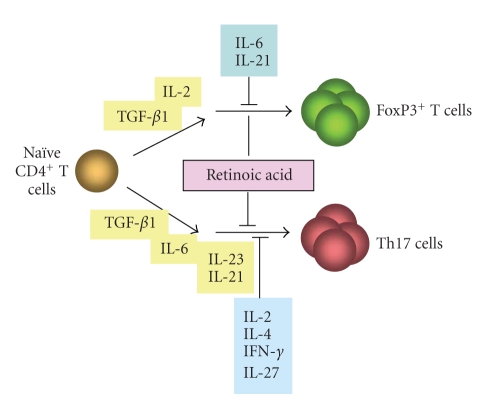
*Reciprocal regulation of* FoxP3^+^ T *cells and* Th17 *cells by retinoic acid.* IL-2 and TGF-*β*1 promote the generation of FoxP3^+^ T cells from naïve T cells in the periphery, while IL-6 and TGF-*β*1 promote the generation of Th17 cells, an inflammatory T cell subset that produces IL-17A, IL-17F, IL-21, and IL-22 as the major effector cytokines. IL-2 and the cytokines that promote T cell proliferation into Th1 or Th2 cells (IL-4, IL-12, IFN-*γ*, and IL-27) would suppress the generation of Th17 cells, while the pro-Th17 cell cytokines, IL-6 and IL-21, can suppress the generation of FoxP3^+^ T cells. Importantly, retinoic acid suppresses the generation of Th17 cells but promotes the induction of FoxP3^+^ T cells.

At-RA, 9-cis RA or other RAR agonists have potent suppressive activities on generation of Th17 cells (Figure 4) [20, 24, 25]. Reciprocally, RAR antagonists can enhance the generation of Th17 cells. In this regard, Elias et al. reported that retinoic acid can suppress ROR-*γ*t expression in T cells [26]. It is paradoxical, however, that Th17 cells are present in the intestine at very high frequencies (~10% of CD4^+^ T cells) in the body [147]. It is likely that the antigen-presenting cells that induce Th17 cells would not be able to produce retinoic acid and are different from the cells that induce gut homing FoxP3^+^ T cells. More studies are required to fully understand the maturation process of Th17 cells in terms of tissue tropism and effector function. Another concern is that most groups used retinoic acid at pharmacological concentrations (~1 *μ*M) to demonstrate the function of retinoic acid in suppression of Th17 cells in vitro. Thus, the in vivo relevance of the function of retinoic acid remains unclear.

## 7. CONCLUSION

Intestine is a large organ that by design is in contact with food digesta, with commensals, and, sometimes, with pathogens. Maintaining the balance between immunity (to pathogens) and tolerance (to food antigens and commensals) is critical to healthy and functional intestine. The recent literature [20–26] supports the view that retinoic acid is an important signal that defines the “microenvironmental cue” of the small intestine. It can be produced by gut epithelial cells and antigen-presenting cells, and it promotes the generation of a gut homing subset of FoxP3^+^ T cells. Since FoxP3^+^ T cells are important for maintaining tolerance in the body, it is likely that retinoic acid is a major signal that confers immunological tolerance to the small intestine. On the other hand, retinoic acid positively regulates also B cell IgA production and induction of gut homing effector T cells, which play essential roles in conferring immunity against pathogens. Thus, we conclude that retinoic acid is a common regulator of both immunity and tolerance in the gut. We still do not completely understand all of the biological functions of retinoid-induced FoxP3^+^ T cells in regulation of immunity and tolerance. Moreover, the readers should be aware of the fact that humans and mice are not identical in this regulation. Also the in vivo relevance of these functions and their impacts on immune responses to pathogens and cancer cells need to be verified in physiological settings. It is also important to investigate if there are additional tissue-specific signals that regulate the generation and migration of FoxP3^+^ and Th17 cells in the gut.
